# The causal nexus between energy consumption, carbon emissions and economic growth: New evidence from China, India and G7 countries using convergent cross mapping

**DOI:** 10.1371/journal.pone.0217319

**Published:** 2019-05-23

**Authors:** Huajun Liu, Mingyu Lei, Naixin Zhang, Guangjie Du

**Affiliations:** 1 School of Economics, Shandong University of Finance and Economics, Jinan, China; 2 Chinese Academy of Finance and Development, Central University of Finance and Economics, Beijing, China; 3 Institute of Finance and Economics Research, Shanghai University of Finance and Economics, Shanghai, China; The Bucharest University of Economic Studies, ROMANIA

## Abstract

Understanding the causality between energy consumption, carbon emissions and economic growth is helpful for policymakers to formulate energy, environmental and economic policies. For the first time, based on nonlinear dynamics, this paper employs multispatial convergent cross mapping (CCM) to revisit the energy-carbon-economy causation for China, India and the G7 countries using both aggregate data and per capita data. The findings indicate that there are significant differences between developing countries and developed countries. A bidirectional nexus between energy consumption, carbon emissions and economic growth is found in China and India, but various causal relationships are identified in the G7 countries, including bidirectional, unidirectional and neutral nexus. The results confirm that the decoupling phenomenon is common in most G7 countries. By leveraging a variety of samples and a new approach, this study provides new evidence for policy authorities to formulate country-specific policies to obtain better environmental quality while achieving sustainable economic growth.

## 1. Introduction

Revealing the precise causal nexus between energy consumption, carbon emissions and economic growth is crucial and imperative for policy authorities to introduce environmentally friendly and economically efficient energy policies. Since the Industrial Revolution, global warming and climate changes caused by rapidly increasing atmospheric levels of CO_2_ and other greenhouse gases (GHGs) have become major concerns worldwide. According to the WMO Greenhouse Gas Bulletin, the rate of increase in atmospheric CO_2_ over the past 70 years has been nearly 100 times greater than that at the end of the last ice age, illustrating an abrupt increasing trend that has never occurred before. In 2016, the atmospheric CO_2_ content reached the highest level in the last 800,000 years [1]. Furthermore, 2015, 2016, and 2017 have been confirmed as the three warmest years on record [2]. Fossil energy consumption is commonly viewed as the major reason for the severe CO_2_ emissions problem, and reducing energy consumption appears to be a necessary step for both developed and developing countries to address the climate change problem. However, due to the accepted view that energy consumption is one of the most important drivers of economic growth [3], the implementation of energy regulation measures has raised major concerns for economic growth. Specifically, if energy consumption causes carbon emissions but is required for economic growth, then adopting energy conservation policies will present many countries with the dilemma of choosing between the “environment or the economy”.

Over the years, many researchers have investigated and revisited the nexus between energy consumption, carbon emissions and economic growth, but no consensus has been reached. Confronted with such confusing and divergent results, many countries have been put in an embarrassing situation when formulating and choosing energy policies. For example, if economic growth does not rely on energy consumption, as specified by the conservation hypothesis (see Kraft and Kraft [4], Menegaki [5] and Rahman and Kashem [6]), then decarbonization-oriented energy conservation policies can be promoted to reduce carbon emissions without worrying about any significant negative effect on economic growth. However, if the energy-economic nexus is consistent with the growth hypothesis (see Yu and Choi [7], Appiah [8], Cai, Sam [9] and Ha, Tan [10]), reducing energy usage may hamper economic growth, which means that policymakers must face the “environment or economy” dilemma. In these cases, alternative energy policies must be designed. Additionally, if economic growth leads to greater carbon emissions, which means that economic growth is achieved at the cost of the environment (see Ang [11], Shahbaz, Mahalik [12] and Mirza and Kanwal [13]), it is necessary to make economic growth more environmentally friendly with lower greenhouse gas emissions. Therefore, precisely understanding the relationship between energy consumption, carbon emissions and economic growth is essential for policy authorities to prudently design appropriate management policies that can help their countries achieve the win-win of the environment and the economy.

According to the conclusions of Karanfil [14] and Ma, Oxley [15], the heterogeneity of recent findings is likely derived from the different methodologies employed, the diverse countries studied, and the different time periods analyzed. With this background, this paper attempt to scientifically identify the causal relationship between energy consumption, carbon emissions and economic growth in China, India and the group of seven (G7 countries) using convergent cross mapping (CCM) for the period 1965–2017. Differing from the existing literature, this study makes significant contributions in two areas: the countries selected and the methodology used.

First, instead of using a sample that only includes a single type of country, this study selects a heterogeneous sample composed of both developed and developing countries. In line with the environmental Kuznets curve (EKC) model, once a threshold economic level is attained, the higher the economic level is, the lower the energy usage and carbon emissions are. Hence, countries in different stages of development might have different causality results. For example, in developed countries, energy consumption may no longer be the cause of carbon emissions and economic growth, but the three may be closely linked in developing countries. Given this, a diverse sample is necessary and useful for country-specific energy policymaking and formulation. Because the G7 is composed of seven of the most industrialized countries in the world (Canada, France, Germany, Italy, Japan, the UK and the US), and China and India are regarded as rapidly developing countries, this study chooses these nine countries as the research sample. Besides, all nine countries are in the top twenty countries in terms of energy consumption and carbon emissions.

Second, based on the nonlinear dynamics perspective, this study extends the CCM approach proposed by Sugihara, May [16] to the field of energy economics. Compared with the Granger causality test, which is commonly used to detect causality between variables, CCM has at least two advantages. First, CCM can be used in nonseparable nonlinear dynamic systems. Nonlinearity is ubiquitous in the real world, and the system composed of energy consumption, carbon emissions and economic growth is no exception [17]. Many studies have considered nonlinearity and have employed the nonlinear Granger causality test (e.g., Hiemstra and Jones [18]), which is usually applicable to separable systems. However, nonlinear dynamic systems are always nonseparable, which means that the Granger causality test may not be applicable. Second, CCM gets rid of control of the model because it is an equation-free approach rather than a model-driven method, such as the Granger causality test. In a way, the data contain all of the information, but the model cannot reflect the entire real world. Therefore, it is inappropriate to model many natural systems with equations [19].

To the best of our knowledge, this is one of the first papers that both employs CCM in energy economics and explores the energy-carbon-economy nexus in different types of countries. The remainder of this paper is organized as follows. Section 2 presents the data and their descriptive statistics. Section 3 introduces the econometric methodology, and Section 4 provides the empirical results and discussion. Section 5 concludes the study and provides policy implications.

## 2. Literature review

Most previous studies on the energy-carbon-economy nexus have focused on the linkage between economic growth and energy consumption or carbon emissions. However, the wide variety of results does not provide consistent solutions for policymakers to balance the relationship between the economy and the environment.

Four main hypotheses for the link between energy consumption and economic growth have been presented in the literature. The first hypothesis, known as the “growth hypothesis” [7], claims that there is a unidirectional causal relationship between energy consumption and economic growth. In this case, any limitation on energy consumption will have a negative impact on economic development [20–22]. The second hypothesis, called the “conservation hypothesis”, was first proposed by Kraft and Kraft [4]. In this situation, there is a unidirectional causal relationship between economic growth and energy consumption, which implies that formulating and implementing conservation energy consumption policies will have no significant adverse influence on economic growth [23, 24]. The third hypothesis is the “neutrality hypothesis” [25], which states that there is no causality between energy consumption and economic growth [5, 26]. The first empirical evidence to support this hypothesis was provided by Akarca and Long [25] and Yu and Hwang [27]. The last hypothesis, known as the “feedback hypothesis”, assumes that there is a bidirectional causal nexus between energy consumption and economic growth. Erol and Yu [28] provided the first empirical evidence for this hypothesis in 5 industrialized countries: Canada, France, Italy, Japan and the UK. This hypothesis indicates that economic outputs and energy usage are mutually determined [8, 10, 29].

The literature about the causality between carbon emissions and economic growth usually builds on the EKC framework. The EKC concept first emerged in the pioneering study conducted by Grossman and Krueger [30] and has since been widely applied in environment economics [31–37]. According to the EKC hypothesis, environmental degradation will transition from an upward trend to a downward trend once the economic level reaches a certain threshold, which has been validated by several studies [38–40]. If carbon emissions increase with economic growth [39–42], economic development still occurs at the expense of the environment, which means that the government needs to make economic growth more environmentally friendly. Conversely, a decrease in carbon emissions with economic growth [12, 38] indicates that economic growth has reached an inflection point and is following a green development path. In addition to the causation from economic growth to carbon emissions, carbon emissions might also have an impact on economic development [13, 39], which indicates that the catch point of the economy could be carbon-related production activities.

Besides, with the increase in clean energy and renewable energy consumption, the relationship between energy consumption and carbon emissions has gradually attracted more attention. Several researchers have found that energy consumption has no significant positive effect on carbon emissions [38, 40], which makes the linkage between energy consumption, carbon emissions and economic growth more complex and confusing.

In fact, the causal nexus between the three factors is a permutation and combination of the relationships between two of the three. The existing literature presents six main relationship loops. In Loop 1, energy consumption has a significant influence on carbon emissions but not on economic growth (see Kraft and Kraft [4], Alper and Oguz [26] and Rahman and Kashem [6]), and carbon emissions can be affected by economic growth (see Ang [11]). Loop 2 is the same as Loop 1 except that energy consumption causes economic growth (see Ang [43]). In Loop 3, energy consumption affects both carbon emissions and economic growth (see Yu and Choi [7] and Appiah [8]), and economic growth can also be influenced by carbon emissions (see Soytas, Sari [44] and Shahbaz, Khraief [45]). Loop 4 is similar to Loop 3, but energy consumption does not cause economic growth (see Menegaki [5]). In Loop 5, energy consumption has no significant impact on carbon emissions, but carbon emissions are a cause of economic growth (see Cai, Sam [9]). Loop 6 is the same as Loop 5 but has a reverse relationship between carbon emissions and economic growth.

## 3. Data and methodology

### Data and its description

**Fig 1:**
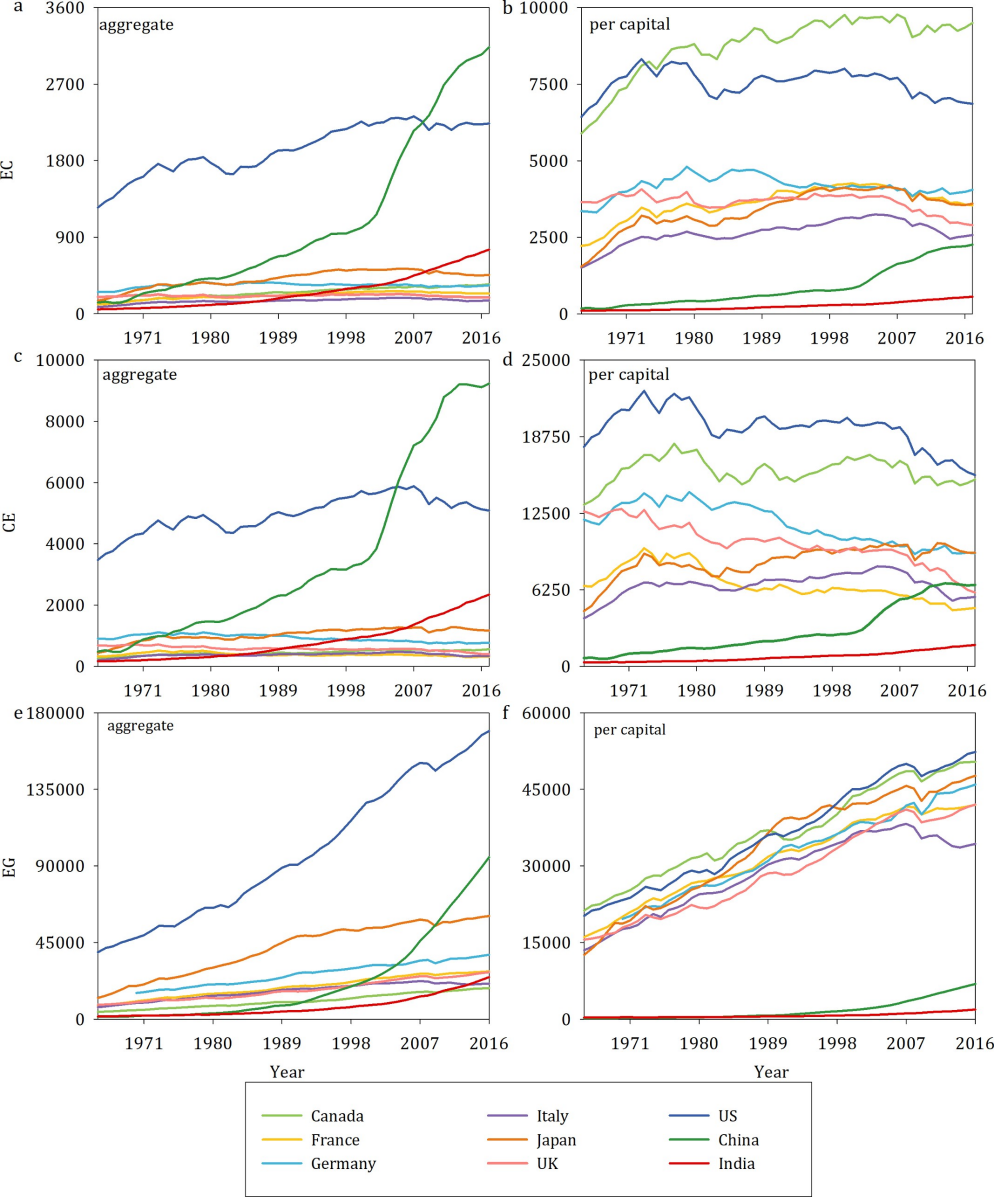
Energy consumption, carbon emissions and economic growth in nine countries. Aggregate energy consumption (a) and per capita energy consumption (b), aggregate carbon emissions (c) and per capita carbon emissions (d), aggregate real GDP (e) and per capita real GDP (f).

Two datasets, aggregate data and per capita data, are used in this study to provide comprehensive results about the causation issue. Both datasets are commonly employed in energy economics research [8, 12, 46]. In the same countries, although the aggregate data have similar trends as the per capita data for all three indexes, the relative levels are significantly different. Fig 1 illustrates these differences. Thus, the causality cannot be investigated using only one dataset.

The annual aggregate data for the nine countries except Germany cover the period 1965–2017, and 1970 is considered the beginning of the economic growth in Germany. Energy consumption (EC) and carbon emissions (CE) are represented in million tons of oil equivalent and million tons of CO_2_, respectively, and are obtained from the BP Statistical Review of World Energy (available at: http://www.bp.com/). Real GDP in constant 2010 prices is acquired from the World Development Indicators (WDI) database and is chosen as a proxy for economic growth (EG). In addition, the total population series from the WDI database are used to transform the three variables into per capita units. Specifically, per capita energy consumption (kg of oil equivalent) and per capita carbon emissions (kg) are denoted as ec and ce, respectively. Economic growth is represented by per capita real GDP in US dollars. Table 1 shows the descriptive statistics for the first dataset. For the EC series, all G7 countries clearly have negative skewnesses. Except for China, India, Germany and Italy, none of the countries have a normal distribution based on the Jarque-Bera statistic. Moreover, only Italy and Germany have kurtosis values larger than 3; the kurtosis is equal to 3 if a series has a normal distribution. Second, for carbon emissions, China, India and France have positive skewnesses, and the kurtosis values for Italy, Japan and the UK are greater than 3. In addition, Canada, Germany, the UK and the US are not normally distributed. Finally, the EG series are negatively skewed only for France, Germany, Italy and Japan. The kurtosis values are generally below 3 for the G7 countries but are greater than 3 for China and India. Moreover, according to the Jarque-Bera test, none of nine countries except for China and India have a normal distribution at the 5% significance level. Table 2 lists the summary descriptive statistics for the second dataset.

**Table 1:** Descriptive statistics for the aggregate data.

	Statistic	China	India	Canada	France	Germany	Italy	Japan	UK	US
EC	Maximum	3132.18	753.70	348.70	266.10	375.40	187.60	530.50	232.30	2320.80
	Minimum	128.53	52.70	115.90	111.30	254.90	78.50	153.30	191.30	1249.60
	Std. Dev.	943.10	203.15	64.52	43.22	26.82	25.42	100.78	11.74	292.70
	Skewness	1.0049	0.9153	-0.3926	-0.7652	-1.4964	-0.9335	-0.7765	-0.1613	-0.4405
	Kurtosis	2.6113	2.7068	2.0756	2.6638	5.3703	3.8255	2.8270	1.9403	2.1682
	Jarque-Bera	9.2544	7.5908	3.2485	5.4213	32.1875	9.2030	5.3920	2.7098	3.2420
	Probability	0.0098	0.0225	0.1971	0.0665	0.0000	0.0100	0.0675	0.2580	0.1977
CE	Maximum	9232.58	2344.20	560.00	515.40	1115.10	472.20	1284.50	718.20	5881.40
	Minimum	475.92	167.70	259.90	304.20	749.40	204.90	446.80	398.20	3480.10
	Std. Dev.	2923.17	630.52	79.94	54.97	112.85	57.86	205.37	73.15	582.19
	Skewness	0.9110	0.9432	-0.4688	0.8357	-0.0302	-0.9326	-1.0243	-0.2714	-0.4789
	Kurtosis	2.3737	2.7799	2.4274	2.8578	1.6842	4.2332	3.6539	3.1634	2.7526
	Jarque-Bera	8.1972	7.9646	2.6658	6.2132	3.8316	11.0410	10.2116	0.7096	2.1608
	Probability	0.0166	0.0186	0.2637	0.0448	0.1472	0.0040	0.0061	0.7013	0.3395
EG	Maximum	101610.10	26295.42	18837.08	28570.89	38657.59	22344.94	61563.29	28069.03	173049.80
	Minimum	1339.23	1618.07	4183.67	8047.79	15340.53	7028.91	12454.70	8452.46	39264.26
	Std. Dev.	28303.04	6734.12	4348.71	6227.26	6937.82	4664.46	15300.35	6054.92	41713.87
	Skewness	1.3867	1.2848	0.2398	-0.0808	-0.0642	-0.4841	-0.4329	0.2327	0.2361
	Kurtosis	3.7252	3.5762	1.8174	1.7508	1.6942	1.9017	1.7269	1.6812	1.6587
	Jarque-Bera	18.1484	15.3142	3.5967	3.5039	3.4433	4.7340	5.2348	4.3190	4.4655
	Probability	0.0001	0.0005	0.1656	0.1734	0.1788	0.0938	0.0730	0.1154	0.1072

**Table 2:** Descriptive statistics for the per capita data.

	Statistic	China	India	Canada	France	Germany	Italy	Japan	UK	US
ec	Maximum	2259.22	562.78	9771.39	4264.15	4804.51	3247.05	4151.58	4070.25	8316.05
	Minimum	167.55	105.94	5890.38	2225.30	3846.21	1507.18	1550.38	2898.26	6431.41
	Std. Dev.	655.53	133.50	979.82	518.92	247.28	394.99	643.51	278.82	440.89
	Skewness	0.9818	0.7651	-1.4401	-1.0278	0.6234	-0.8509	-0.9360	-1.2945	-0.4292
	Kurtosis	2.5679	2.4713	4.3180	3.6843	2.3386	3.8943	3.5240	3.9363	2.3666
	Jarque-Bera	8.9280	5.7880	22.1558	10.3649	3.9840	8.1611	8.3460	16.7389	2.5130
	Probability	0.0115	0.0554	0.0000	0.0056	0.1364	0.0169	0.0154	0.0002	0.2847
ce	Maximum	6780.88	1750.50	18180.00	9644.00	14236.70	8165.90	10064.40	12850.50	22483.20
	Minimum	615.46	335.20	13205.40	4586.60	9169.60	3931.30	4518.20	6030.90	15619.8
	Std. Dev.	2016.19	415.33	1069.83	1341.07	1704.84	936.82	1232.90	1678.19	1615.66
	Skewness	0.8939	0.7976	-0.2767	0.5919	0.0482	-0.8364	-1.3930	-0.3174	-0.5435
	Kurtosis	2.3509	2.5469	2.7609	2.4613	1.4170	3.7111	5.0311	2.8585	3.0270
	Jarque-Bera	7.9885	6.0725	0.8027	3.7356	5.0306	7.2960	26.2514	0.9339	2.6112
	Probability	0.0184	0.0480	0.6694	0.1545	0.0808	0.0260	0.0000	0.6269	0.2710
eg	Maximum	7329.09	1963.55	51315.89	42567.74	46747.19	38236.80	48556.93	42514.49	53128.54
	Minimum	172.91	318.4157	21260.63	16087.92	19624.75	13487.99	12595.39	15552.46	20207.75
	Std. Dev.	2039.58	452.98	9000.87	8096.89	8036.18	7470.14	10767.99	8785.66	10292.38
	Skewness	1.3397	1.2534	-0.0087	-0.3017	-0.0634	-0.5083	-0.4412	0.0455	0.0170
	Kurtosis	3.5805	3.4598	1.7972	1.8509	1.7971	1.9271	1.7713	1.5651	1.6005
	Jarque-Bera	16.5989	14.3436	3.1955	3.7200	2.9261	4.8240	5.0531	4.5650	4.3281
	Probability	0.0002	0.0008	0.2024	0.1557	0.2315	0.0896	0.0799	0.1020	0.1149

### Convergent cross mapping

#### Basic theorem of CCM

CCM was introduced based on the state space reconstruction (SSR) technique, which is an advanced nonparametric technique developed by Sugihara, May [47]. Since the embedding theory was introduced by Takens [48], the SSR technique has become a favorable framework for developing nonlinear methods to detect the interactions between nonlinear time series. Based on the simplex projection proposed by Sugihara and May [49], CCM has been developed and applied to nonlinear dynamic systems to investigate the correspondence between two variables by using time-delayed embedding to reconstruct two shadow manifolds of *X* and *Y*.

If two variables *Y* and *X* are dynamically coupled, then they share a collaborative attractor manifold *M*, which is a d-dimensional manifold in an *E*-dimensional state space (*d* ≤ *E*), and *X* and *Y* can be used to reconstruct their own shadow attractor manifolds *M*_*X*_ and *M*_*Y*_. Accordingly, CCM is employed to evaluate the causality between *X* and *Y* by determining how precisely the attractor manifold *M*_*X*_ corresponds to *M*_*Y*_.

#### Essential concept of CCM

For observations, the response process is better than the forcing process, which is the essential concept of CCM. Specifically, in dynamic systems, the response process contains information about the forcing process, but the forcing process is likely not the dominant predictor of the response process. Videlicet, CCM and its derivative approaches explore reasonable methods to evaluate the causal interactions between *X* and *Y* by using the historical record of *Y* values to estimate the states of *X* variables. To determine whether *X* causally influences *Y*, the idea of CCM is to confirm whether it is accurate to leverage the attractor manifold *M*_*Y*_, built from lagged coordinates of time series *Y*, to identify nearby points of *M*_*X*_ constructed from the *X* variable. If it is, then the *Y* series can be used to estimate *X*, and vice versa.

#### An algorithm for CCM

Consider two time series of length *L*, *X* and *Y*, which are represented as follows:

{Y}={Y(1),Y(2),Y(3),…,Y(L)} (1)

By forming the lagged coordinate vectors of *X* and *Y*, the shadow attractor manifold *M*_*X*_ and *M*_*Y*_ can be reconstructed as follows:

$$\begin{array}{l} \underline{x}\left(t\right)=<X\left(t\right),X\left(t-\tau \right),X\left(t-2\tau \right),\ldots ,X\left(t-\left(E-1\right)\tau \right)>\\ \underline{y}\left(t\right)=<Y\left(t\right),Y\left(t-\tau \right),Y\left(t-2\tau \right),\ldots ,Y\left(t-\left(E-1\right)\tau \right)> \end{array}$$ (2)

where t = 1 + (E − 1)τ to *t* = *L*, *E* represents the embedding dimension, which describes the size of the time window, and the time lag *τ* is positive. From the first lagged coordinate *X*(*t*) to the last, *X*(*t* − (*E* − 1)*τ*), there are *E-*dimensional points. According to the results of Sugihara and May [49], the smallest bounding simplex is formed from the *E* + 1 closest neighbors, such that the simplex can contain *E*-dimensional points. Thus, there is an essential numerical limit on the potential embedding dimension *E*. If there are *n* observations, then *E*≤*n*-1, as the analysis requires *n* − 1 observations to describe the historical dynamics and at least one observation to evaluate the estimated values [50].

Since *My* is diffeomorphic from *Mx*, the time indexes corresponding to the nearest neighbors to $\underline{x}(t)$ on *M*_*X*_ can be used to estimate *Y*(*t*) by creating a cross mapping of *Y*(*t*) from a locally weighted mean of *E* + 1 *Y*(*ti*) values. The cross-mapped estimation of *Y*(*t*) is denoted by $\hat{Y}(t)|M_{X}$, which represents using *M*_*X*_ to estimate *Y*(*t*) to detect the causal relationship from *Y*→*X*, and the cross mapping from *Y* to *X* is defined analogously. In particular, $\hat{Y}(t)|M_{X}$ can be calculated as follows:

$$\hat{Y}\left(t\right)|M_{X}=\sum w_{i}Y\left(t_{i}\right)\;i=1,2\ldots ,E+1$$ (3)

where *w*_*i*_ is the weighting calculated by the distance between $\underline{x}$ and its *i*^th^ neighbor on *M*_*X*_, and *Y*(*t*_*i*_) are the values of Y; the formula for the weighting is as follows:

$$w_{i}=u_{i}/\sum u_{j}\;j=1,2\ldots ,E+1$$ (4)

where

$$u_{i}=\exp\left(-\frac{d\left[\underline{x}\left(t\right),x\left(t_{i}\right)\right]}{d\left[\underline{x}\left(t\right),x\left(t_{1}\right)\right]}\right)$$ (5)

where $d[\underline{x}(s),x(t)]$ indicates the Euclidean distance between two vectors.

Based on the cross mapping, a library consisting of *L* points from *M*_*X*_ can be used to provide estimates of *L* points for the original time series *Y*. Then, the Pearson correlation coefficient $\rho_{Y{\hat{Y}}_{t}}$, which represents the ability of the *L* cross-mapped estimates from *X* to describe the *L* true value from *Y*, can be calculated. Specifically, the CCM correlation is determined as follows:

$$\rho_{Y{\hat{Y}}_{t}}=\rho \left(Y\left(t\right),\hat{Y}\left(t\right)|M_{X}\right)$$ (6)

Given that the response process contains information about the forcing process, the Pearson correlation coefficient $\rho_{Y{\hat{Y}}_{t}}$ is an indicator for asserting the intensity of the effect from *Y* to *X*. Thus, if *Y* has a causal impact on *X*, then $\hat{Y}(t)|M_{X}$ will converge to *Y*(*t*), and $\hat{X}(t)|M_{Y}$ should converge to *X*(*t*); that is, the CCM correlation should increase to *L* until infinity. In reality, the forecast skill of the cross-map estimates from *X* to *Y* can only reach a plateau.

#### Two improvements of multispatial CCM

Although CCM provides a relatively good methodology by using a simplex projection, it still requires a relatively long time series. Accordingly, the first extension performed by multispatial CCM is to mend the simplex projection. Because spatially replicated data involve temporal information and replication seems plausible to compensate for the sententiousness of time series [51], Clark, Ye [50] proposed multispatial CCM as a novel approach. By combining CCM and the bootstrapping technique [51], multispatial CCM successfully leverages as few as five observations.

Another extension performed by multispatial CCM involves incorporating iteration into the estimation procedure. Multispatial CCM repeats the CCM algorithm for n samples that are drawn from n spatial replicates. It should be noted that the number of iterations must commonly exceed 100 [50]. By repeating the estimation procedure with sufficient iterations, abundant potential combinations and orders of spatial data can be averaged, and the uncertainty over the predictive power of each test can be estimated more accurately.

## Empirical results and discussion

### A detailed multispatial CCM example

**Fig 2:**
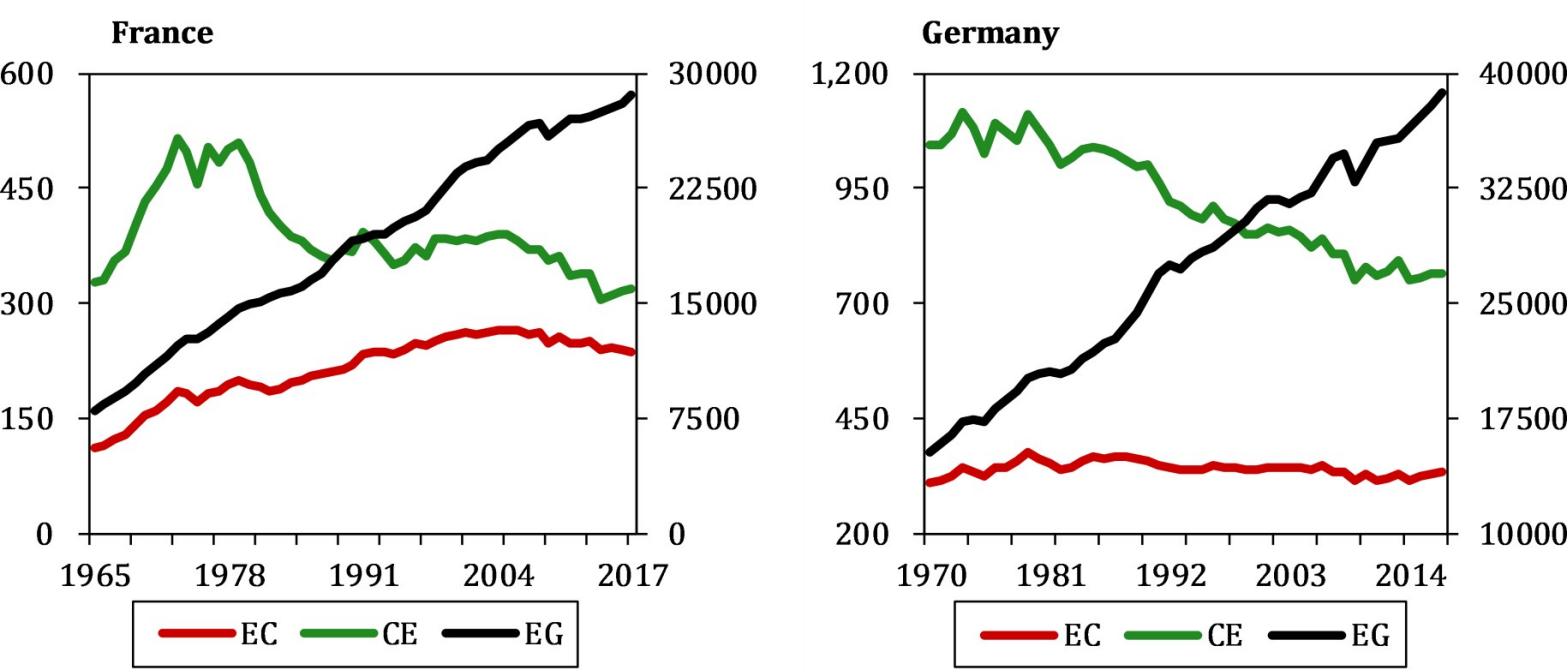
The temporal trends of EC, CE and EG for France and Germany.

Multispatial CCM repeatedly implements complete steps for many iterations to demonstrate the significant causal relationship between energy consumption, carbon emissions and economic growth. The procedure can be divided into three steps: choosing an optimal embedding dimension *E*, checking the nonlinearity of the candidate time series and applying the CCM algorithm. Furthermore, three causations can be summarized from the results identified by CCM: bidirectional, unidirectional and neutral causalities. Because the results for France and Germany embody all three possible scenarios, this study used these two countries as examples to demonstrate the complete CCM procedure. Fig 2 shows the temporal trends of energy consumption, carbon emissions and economic growth for France and Germany.

#### Determining the embedding dimension

To perform SSR to generate the shadow manifolds, the most important step is to determine an optimal E for each time series, and the best turning parameter E can then be used to form the lagged coordinate vectors for each candidate variable. The relation between the forecast skill of each time series and E can be used to select the best E. Specifically, by employing the univariate simplex projection technique, each candidate effect series can be calculated as a correlation coefficient ρ between the predicted and observed results, which is shown as a function of E for the predictions that are one time step in the future. Moreover, the correlation coefficient can be plotted to intuitively indicate the changes in predictive power with respect to a different embedding dimension E. Therefore, the basic principle of optimizing the parameter E for each time series is to choose one embedding dimension E that corresponds to the most accurate prediction; i.e., the E where the peak ρ is located [49].

**Fig 3:**
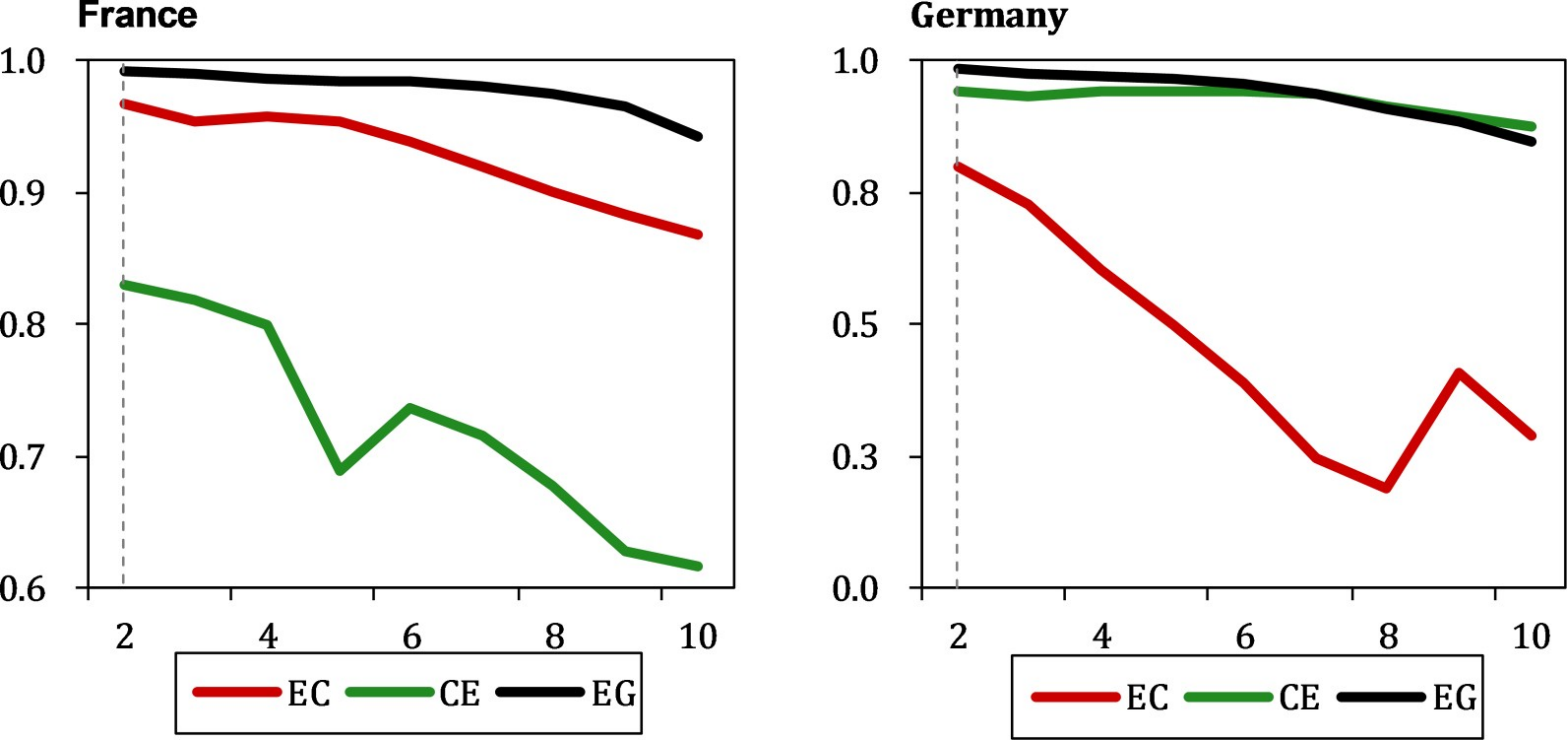
Determining the optimal E of EC, CE, EG for France and Germany.

The variations in the coefficient ρ between the predicted and observed results with an increase in the embedding dimension E are illustrated in Fig 3. Clearly, the maximum predictive power occurs when the optimal E equals 2. In addition, the time delay τ is set to 1 based on the average mutual information criterion [52].

#### Checking the nonlinearity of the time series

Multispatial CCM can give misleading results when it is employed in a purely linear system in which the nonlinear structure is masked. Therefore, it is necessary to check for the presence of nonlinear structures for each candidate time series using the univariate simplex projection. According to Clark, Ye [50], in the case of nonlinearity, the forecast skill should decrease with increasing step size, meaning that the prediction is more accurate for short time intervals than for long time intervals. Conversely, if a periodicity exists in the system dynamics that causes the predictive power to form a U-shaped curve, or if noise or cyclic factors exist in the data, leading to a few spikes after the decrease in predictive power, then a result of nonlinearity is inconclusive [50].

**Fig 4:**
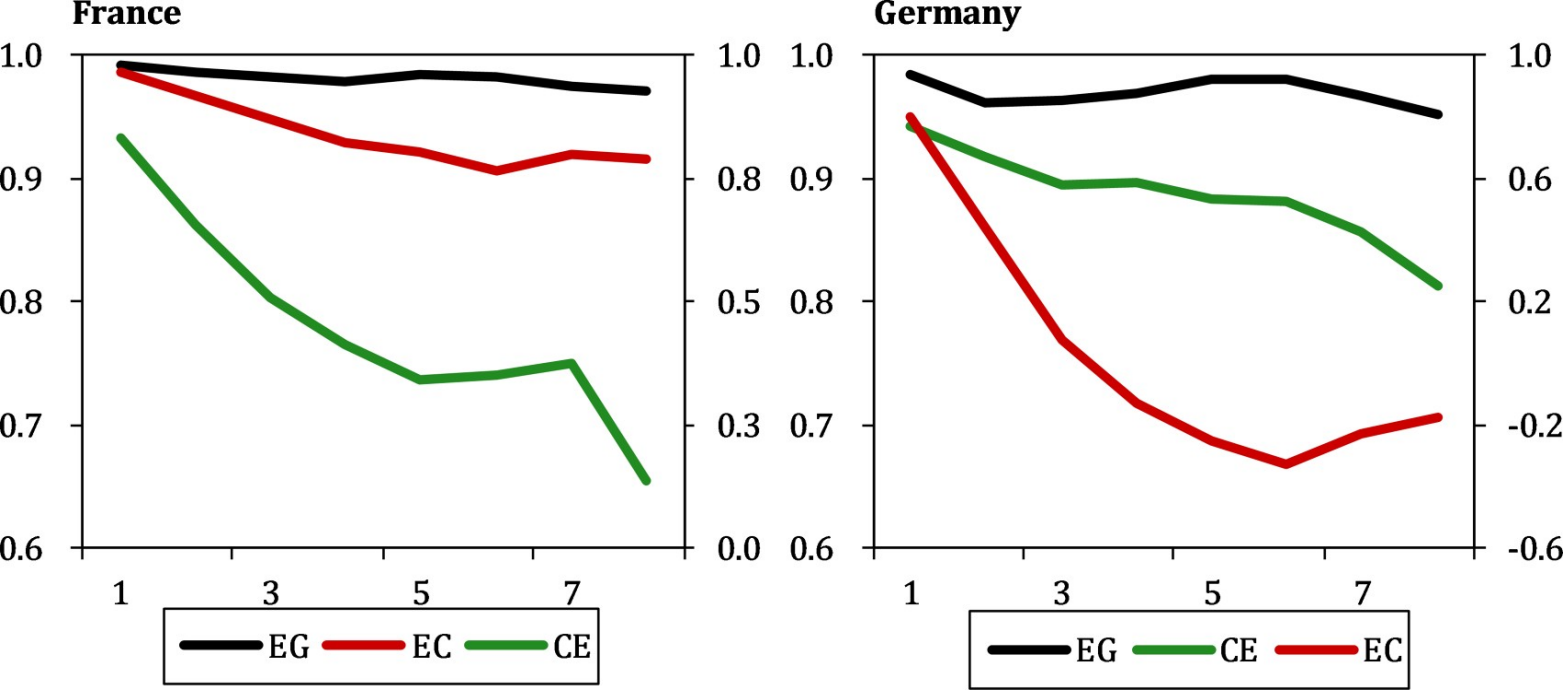
The nonlinearity of EC, CE, EG for France and Germany.

Stern and Enflo [17] confirm the presence of nonlinearity in energy and output, and this result is verified in this study. Fig 4 shows the nonlinearity tests for the three indexes for France and Germany. Both time series have significant decreasing trends.

#### Results of multispatial CCM

**Fig 5:**
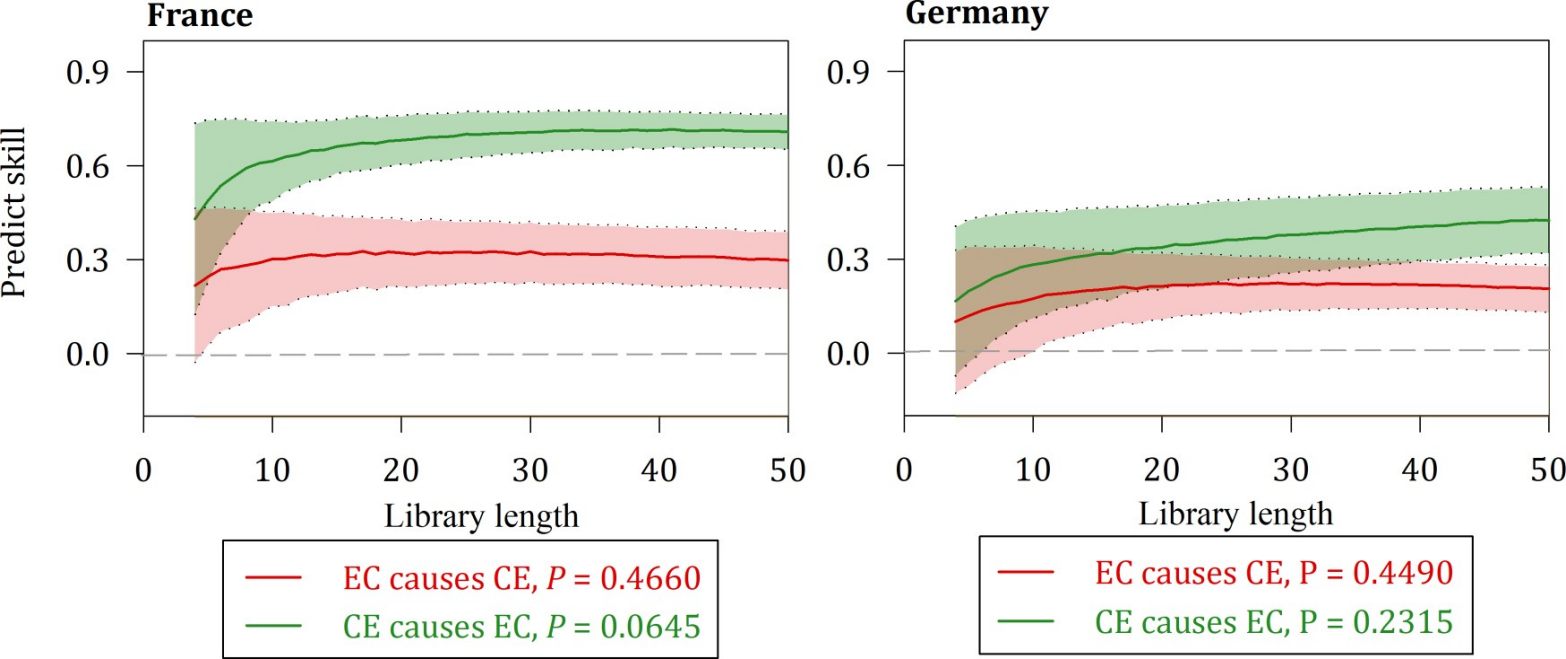
Causality between EC and CE for France and Germany.

**Fig 6:**
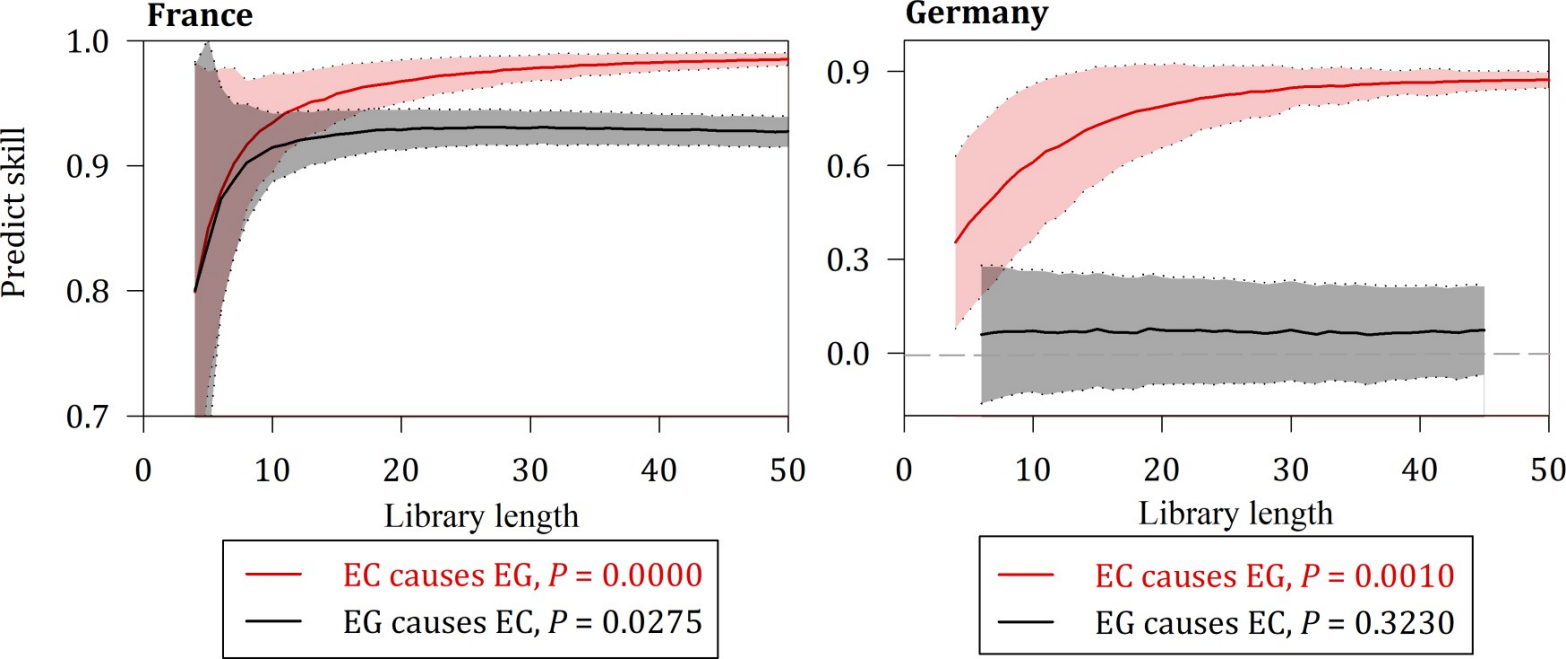
Causality between EC and EG for France and Germany.

**Fig 7:**
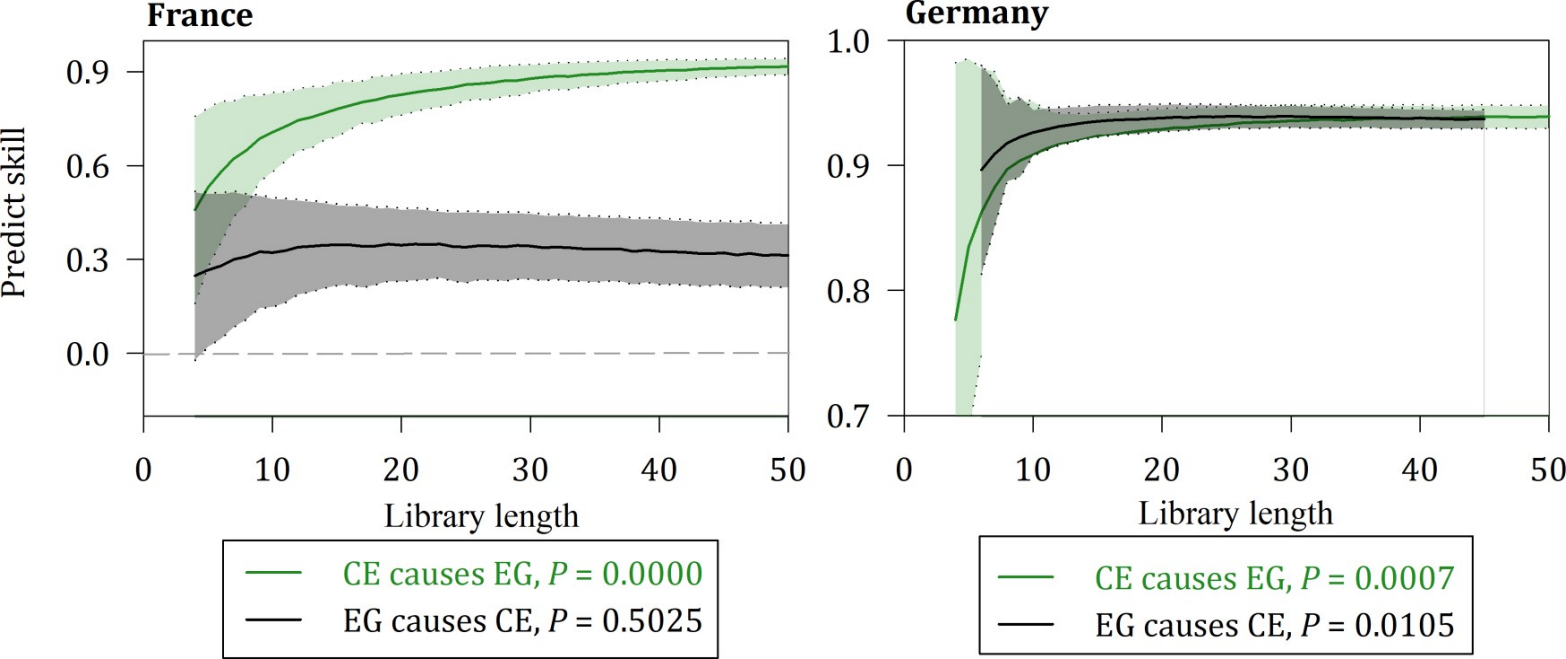
Causality between CE and EG for France and Germany.

Figs 5, 6 and 7 suggest a causal relationship demonstrated by the multispatial CCM between the three variables for France and Germany.

The solid lines show the means of the predictive power, and the shaded regions represent the mean plus or minus its standard deviation. All three cases of causality are contained in the two nations at the 10% significance level.

### Causality between EC, CE and EG for nine countries: Evidence from aggregate data

The CCM results for the nine countries are reported in Table 3. From the perspective of the causation between EC and CE, the results indicate that six nations have a bidirectional nexus: China, India, Canada, Italy, Japan and the US. Germany and the UK have neutral causalities, while France has a unidirectional causality from CE to EC.

**Table 3:** Multispatial CCM test for the aggregate data.

Hypothesis	EC—CE			EC—EG			CE—EG		
	EC→CE	CE→EC	direction	EC→EG	EG→EC	direction	CE→EG	EG→CE	direction
China	0.0000***	0.0000***	Two-way	0.0005***	0.0000***	Two-way	0.0000***	0.0000***	Two-way
India	0.0000***	0.0000***	Two-way	0.0000***	0.0000***	Two-way n	0.0000***	0.0000***	Two-way
Canada	0.0025***	0.0000***	Two-way	0.0000***	0.0000***	Two-way	0.0000***	0.0145**	Two-way
France	0.4660	0.0645*	CE→EC	0.0000***	0.0275**	Two-way	0.0000***	0.5025	CE→EG
Germany	0.4490	0.2315	Neutrality	0.0010***	0.3230	EC→EG	0.0070***	0.0105**	Two-way
Italy	0.0045***	0.0000***	Two-way	0.0000***	0.0090***	Two-way	0.0010***	0.104	CE→EG
Japan	0.0015***	0.0025***	Two-way	0.0000***	0.0035***	Two-way	0.0005***	0.036**	Two-way
UK	0.1615	0.3955	Neutrality	0.0235**	0.4200	EC→EG	0.0045***	0.1325	CE→EG
US	0.0000***	0.0000***	Two-way	0.0000***	0.0225**	Two-way	0.0000***	0.0670*	Two-way

*, **, and *** denote the 1%, 5% and 10% significance levels, respectively.

For the causal nexus between EC and EG, the results indicate that two-way causality exists in all of the countries except Germany and the UK, which have unidirectional causal relationships from EC to EG. Clearly, energy is still a key element of economic growth in most of the G7 countries except Germany and the UK. For these two countries, energy consumption can still stimulate economic growth, but the economy can accommodate some development without additional energy consumption; this means that energy is no longer the dominant factor of economic growth.

For the causality between CE and EG, the outcomes in six countries (i.e., China, India, Canada, Germany, Japan and the US) illustrate that the two indexes are jointly determined and mutually influencing, whereas France, Italy and the UK have unidirectional causality from carbon emissions to economic growth, in contrast to the EKC hypothesis. The unidirectional causality from carbon emissions to economic growth for France, Germany and the UK implies that high-emissions economic activities can promote economic growth, but development can be achieved using other decarbonization production activities, which means that economic growth is gradually decoupling from carbon emissions.

**Fig 8:**
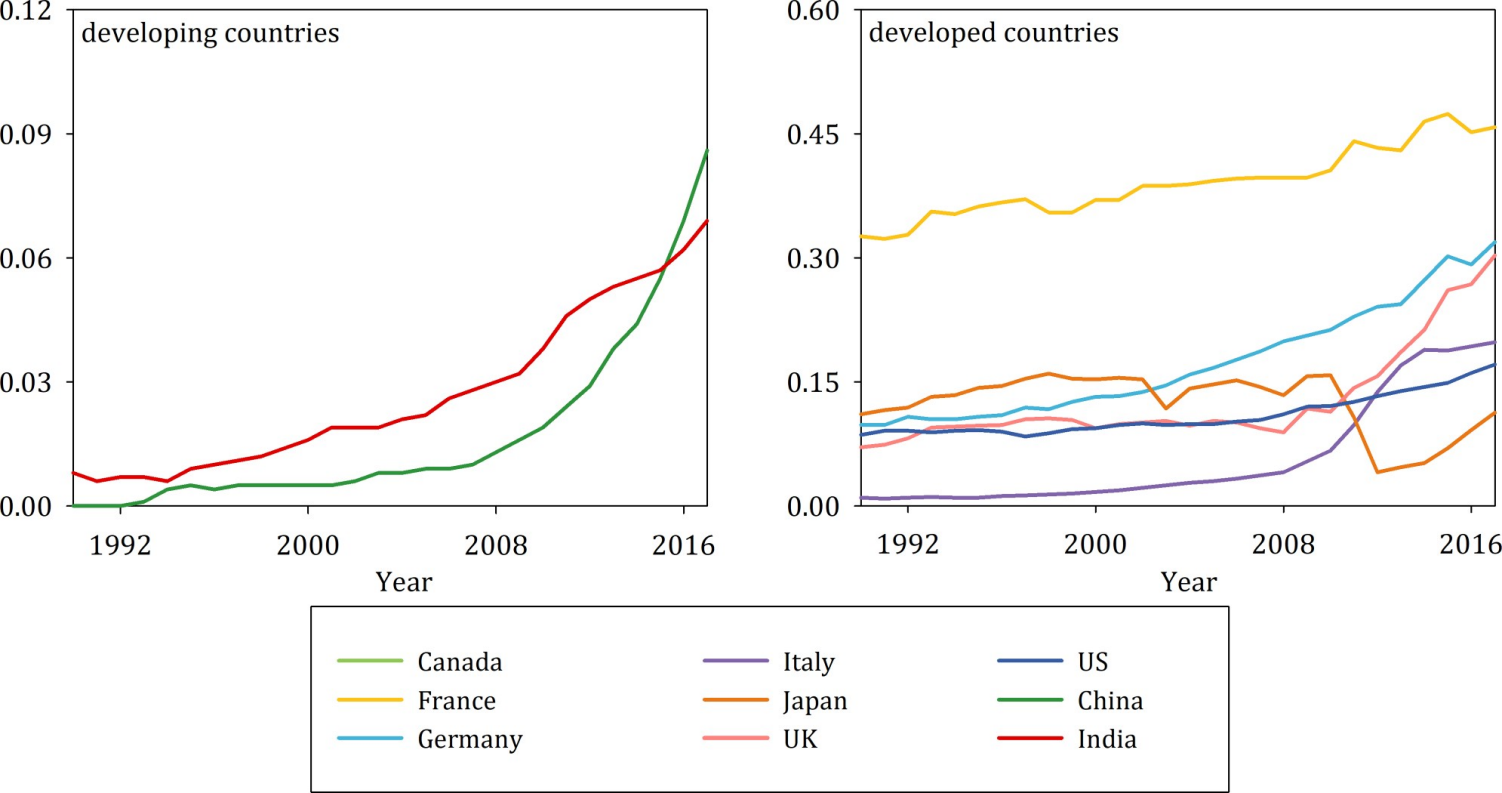
Clean energy as a proportion of primary energy.

A comparison between the developing countries (represented by China and India) and developed countries (represented by the G7 countries) shows intense causal linkages between energy consumption, carbon emissions and economic growth in most of the countries, but there are differences in several of the G7 countries. Specifically, in France, Germany and the UK, these three variables explicitly signal a gradual decoupling. Given that France, Germany, Italy, Spain, and the UK (namely, the EU-5 countries) share a common 20/20/20 objective, this study reasonably speculates that the transformation of the energy structure can be considered a critical reason for the presence of the decoupling phenomenon. The data shown in Fig 8 can be used to analyze this hypothesis. The proportion of primary energy provided by clean energy commonly exceeds 10% for the G7 countries, and it exceeds 30% in France, Germany and the UK.

However, as developing countries, China and India still have lower levels of clean energy (less than 10%). Coincidentally, the four countries with the highest clean energy consumption are those nations in which the decoupling phenomenon is observed. The transmission mechanism from clean energy consumption to other variables can be described as follows. With increasing clean energy consumption, the energy consumption of economic activities is no longer the “sufficient condition” of carbon emissions, causing energy consumption to decouple from carbon emissions. Clean energy consumption still facilitates economic growth with low levels of CO_2_ emissions, which explains the presence of a decoupling nexus between economic growth and carbon emissions.

In addition, developed countries have higher energy usage and more advanced production technology and low-carbon technology than developing countries, which might also be a cause of the decoupling phenomenon.

### Causality between EC, CE and EG for nine countries: Evidence from per capita data

It is reasonable to address the same question using per capita data, given the different information contained by the two datasets. Table 4 summarizes the results derived from the per capita data. Clearly, China and India still have strong causal linkages between the three variables, but decoupling is found in most of the G7 countries.

**Table 4:** Multispatial CCM test for per capita data.

Hypothesis	ec—ce			ec—eg			ce—eg		
	ec→ce	ce→ec	direction	ec→eg	eg→ec	direction	ce→eg	eg→ce	direction
China	0.0000***	0.0000***	Two-way	0.0000***	0.0000***	Two-way	0.0000***	0.0000***	Two-way
India	0.0000***	0.0000***	Two-way	0.0000***	0.0005***	Two-way	0.0000***	0.0000***	Two-way
Canada	0.194	0.0945*	ce→ec	0.0005***	0.1465	ec→eg	0.0245**	0.328	ce→eg
France	0.2055	0.122	Neutrality	0.0005***	0.0425**	Two-way	0.0005***	0.062*	Two-way
Germany	0.6975	0.495	Neutrality	0.0005***	0.668	ec→eg	0.0105**	0.0455**	Two-way
Italy	0.0005***	0.0025***	Two-way	0.0015***	0.045**	Two-way	0.001***	0.171	ce→eg
Japan	0.01**	0.0125**	Two-way	0.0005***	0.011**	Two-way	0.001***	0.1405	ce→eg
UK	0.0435**	0.1515	ec→ce	0.244	0.1365	Neutrality	0.0905*	0.008***	Two-way
US	0.001***	0.0165**	Two-way	0.006***	0.177	ec→eg	0.1445	0.0215**	eg→ce

*, **, and *** denote the 1%, 5% and 10% significance levels, respectively.

The bidirectional causality between the three variables for China and India are the same as the results from the aggregate data. This fact indicates that economic growth in these two countries might come at the cost of carbon emissions and that fossil energy consumption is still considered a key driver. The findings for the G7 countries are slightly different from the results obtained from the aggregate data. Clearly, the decoupling phenomenon becomes more general in the G7 countries. With respect to the nexus between energy consumption and carbon emission, three countries–Italy, Japan and the US–have bidirectional causalities, and France and Germany verify the neutrality hypothesis. Carbon emissions have a causal effect on energy consumption in Canada, but the UK has the opposite relationship. For the causality between energy consumption and economic growth, there are three cases of a bidirectional causal nexus, namely, France, Italy and Japan. Three countries, Canada, Germany, and the US, have unidirectional causalities from energy consumption to economic growth. Furthermore, the UK has a neutral causality, which is considered a completely decoupled relationship. Finally, for the causality between carbon emissions and economic growth, feedback effects are found in France, Germany and the UK. Canada, Italy, and Japan have unidirectional effects from carbon emissions to economic growth, and the US shows causality in the opposite direction.

## Conclusions and policy implications

This paper investigates the causal nexus between energy consumption, carbon emissions and economic growth for China, India and the G7 countries using a novel approach, multispatial CCM, that was proposed by Clark, Ye [50]. To comprehensively address this question, this study uses two datasets consisting of aggregate data and per capita data. After determining the optimal E, checking the nonlinearity of the time series and performing the CCM, the results illustrate significant differences between developing countries (i.e., China, India) and developed countries (i.e., the G7 countries). The findings suggest that there are strong causal linkages between these three variables in China and India. However, a decoupling nexus is found in most of the G7 countries. The empirical results are helpful for policymakers in formulating energy, environmental, and economic policies to optimize better living conditions while fostering sustainable economic growth.

The bidirectional causal nexus between energy consumption and economic growth in China and India implies that energy consumption not only drives economic growth but also is crucial for the economy. The results indicate that both the Chinese and Indian governments must be cautious in implementing energy conservation policies in the short term because greatly reducing the energy supply will have an adverse impact on economic growth. In addition, the feedback causal nexus between carbon emissions and economic growth suggests that development still comes at the cost of environmental quality, which requires policymakers to seek a green development path quickly. Reductions in energy consumption can be regarded as an important instrument to decrease CO_2_ emissions because there is a bidirectional causality between energy consumption and CO_2_ emissions. Thus, to achieve sustainable development while reducing carbon emissions, it is necessary for policy authorities in China and India to seek substitute energy sources.

A unidirectional causality between the three variables is found in Canada with the per capita data, indicating that energy consumption is a driver of economic growth but not the only driver, and enhancing economic growth might no longer come at the cost of environment. These results imply that formulating energy conservation policies to reduce energy consumption might have a harmful influence on economic growth in Canada, but the impact will not lead to fatal consequences. However, to decrease carbon emissions, Canada needs to find other solutions because a reduction in energy consumption has no causal effect on carbon emissions, which is possibly due to the increasing usage of clean energy. Italy and Japan have bidirectional causalities between energy consumption and economic growth and between energy consumption and carbon emissions as well as a unidirectional causal nexus from carbon emissions to economic growth. The results for Italy and Japan indicate that energy consumption remains a pillar of economic growth but that economic development is becoming more environmentally friendly. Therefore, policymakers in Italy and Japan should enhance environmental governance, adopt energy-efficient technology, increase the demand for clean energy, and seek other green economic policies to stimulate increased output.

The decoupling phenomenon was identified in France and Germany, the two largest consumers of clean energy. In France, there is a bidirectional causality between energy consumption and economic growth regardless of the dataset used. Moreover, a unidirectional nexus from carbon emissions to energy consumption can be found in the aggregate data, but a neutral nexus is found in the per capita data. The findings for France indicate that energy consumption remains a key driver of economic growth but will not increase carbon emissions due to the significant clean energy usage. In addition, it is difficult to determine whether economic growth comes at the cost of the environment given the controversial results derived from the two datasets. Therefore, it is necessary for France to implement a fossil energy conservation policy, enhance the efficiency of energy use, and expand the demand for clean energy to maintain economic growth while decreasing atmospheric pollution. In Germany, there is no difference between the results from the two datasets. A unidirectional causality runs from energy consumption to economic growth, implying that energy conservation policies may not generate a large shock to growth. No causality is found between energy consumption and CO_2_ emissions, which suggests that energy consumption is no longer the main cause of air pollution. The feedback causality between CO_2_ emissions and economic growth reveals that growth in Germany still comes at the cost of the environment, but the emissions source is not energy consumption. This situation requires policymakers to determine the true cause of carbon emissions and adopt policies of environmental governance and protection.

The results for the UK indicate that energy consumption plays a minor role in stimulating economic growth, supporting the implementation of energy conservation policies. However, economic growth might still come at the cost of carbon emissions, and the usage of energy can result in increased carbon emissions, revealing the demand for environmental governance policies, clean energy and emissions reduction mechanisms. The empirical findings for the US imply that there are unidirectional causalities from energy consumption to economic growth and from economic growth to carbon emissions. In addition, there is a bidirectional causality between energy consumption and carbon emissions. The results indicate that the US might still be following an energy-driven and environment-consuming development path. Therefore, it is urgent for the US to facilitate energy-efficient emissions reduction technologies and to enhance environmental governance efforts when accelerating economic growth.

## Supporting information

S1 File Processed data. (ZIP)
